# Epidemiology of fungal diseases in Africa: A review of diagnostic drivers

**DOI:** 10.18502/cmm.7.1.6246

**Published:** 2021-03

**Authors:** Felix Bongomin, Samuel Adetona Fayemiwo

**Affiliations:** 1 Department of Medical Microbiology and Immunology, Faculty of Medicine, Gulu University, Gulu, Uganda; 2 Department of Medical Microbiology and Parasitology, College of Medicine, University of Ibadan, University Hospital Ibadan, Ibadan, Nigeria; 3 Division of Infection, Immunity and Respiratory Medicine, Faculty of Biological Sciences, University of Manchester, Manchester, UK

**Keywords:** Aspergillosis, Candidiasis, Cryptococcosis, Emergomycosis, Fungal disease, Mycetoma

## Abstract

**Background and Purpose:**

There has been a significant increase in the burden of fungal diseases in the last few decades which has imposed a global threat to the health of humans, animals, and plants. Epidemiology of fungal diseases is not completely understood in Africa. Most of these diseases are under-reported or not reported at all mainly due to the challenges related to the availability of and access to fungal diagnostics and the lack of human resources in clinical and diagnostic mycology across the continent. Therefore, it is imperative to highlight the epidemiology of the endemic and epidemic of emerging and re-emerging fungal diseases as well as their diagnostic challenges in Africa based on the available data. Moreover, it is important to underline the existing gaps in this regard as well.

**Materials and Methods:**

For the purposes of the study, Medline and Google Scholar were searched to retrieve articles on these prominent fungal diseases, as well as their etiologies and available diagnostics.

**Results:**

It was found that histoplasmosis and other AIDS-associated mycoses have been reported in Africa, including blastomycosis, coccidioidomycosis, and paracocci-dioidomycosis. Other reported infections were fungal neglected tropical diseases, especially sporotrichosis, dermatophytosis, mycetoma, and chromoblastomycosis as well as emerging fungal diseases, such as *Emergomyces africanus*, *Candida auris*, and *Blastomyces emzantsi*. In Africa, the major drivers of fungal diseases include human immunodeficiency infection, tuberculosis, and poverty.

**Conclusion:**

Serious fungal diseases are common in Africa; however, the true burden remains unknown.

## Introduction

In the last few decades, there has been a significant increase in the burden of fungal diseases which continues to pose a global threat to the health of humans, plants, and animals [ 1 ]. More than a billion fungal diseases with variable severity occur every year worldwide [ 2 ]. These fungal infections include dermatophytosis, vulvovaginal candidiasis, allergic diseases, subcutaneous infections, and life-threatening invasive systemic diseases [ 2 ]. Over 300 fungal species cause human diseases, mainly in the immunocompromised individuals and those with a competent immune system, especially people who have underlying chronic respiratory diseases and co-morbidities or live in poverty [ 3 ].

Main drivers of fungal diseases in Africa include the human immunodeficiency (HIV) and tuberculosis (TB) syndemic, poverty, and the growing number of individuals with non-communicable diseases, notably cancer, asthma, and diabetes mellitus. It is noteworthy that > 25 million (~70%) out of the estimated 37.9 million people living with HIV (PLHIV) worldwide are in Africa [ 4 ]. 

Despite the popularity of antiretroviral therapy (ART), cryptococcosis, pneumocystosis, histoplasmosis, and other opportunistic fungal infections continue to threaten the lives of PLHIV, particularly those who present with advanced HIV disease or failed their ART [ 4 , 5 ]. Moreover, the fungal neglected tropical diseases (FNTD), notably mycetoma, chromoblastomycosis, sporotrichosis, and dermatophytosis occur in areas with poverty. 

The spectrum of fungal diseases has posed a heavy burden on Africa which differs in one way or the other from the global statistics. However, the epidemiology of fungal diseases in Africa is not understood completely. This is mainly due to the challenges related to the availability of and access to fungal diagnostics and the lack of human resources in clinical and diagnostic mycology across the continent. 

Definitive diagnosis of fungal disease requires confirmation of the causative pathogen through culture and microscopy, histopathology, immunodiagnostics, biomarkers, molecular assays, and advanced molecular detection, alone or in combination. In many parts of Africa, this is practically hampering accurate evaluation of the incidence of fungal infections.

As practicing mycologists in Africa, we are faced with several endemic, epidemic, emerging, and re-emerging fungal diseases requiring correct identification and appropriate treatment. This article aimed to highlight the key fungal diseases, their methods of diagnosis, and the challenges in Africa. 

## Materials and Methods

### Literature review

The related literature was found in Medline (through PubMed) and Google Scholar. In total, four search strategies were used to retrieve the articles: 1) each of the fungal diseases was searched, such as “cryptococcosis”, “emergomyces”, “chronic pulmonary aspergillosis”, and “Madura foot” plus “Africa”, 2) etiologic fungal genus or species were searched, namely “*Aspergillus*”, “*Emergomyces africanus*”, and “*Histoplasma capsulatum*” plus “Africa”, 3) “Africa” was also replaced with each of the individual 54 countries of Africa, and 4) the words used in the first three strategies were combined with the word “diagnostics”. Alternative names or syndromes of each of the disease entities were used as well, such as cryptococcosis, cryptococcal meningitis, or asymptomatic cryptococcaemia. 

The related literature in English was reviewed and there was no restriction on the year of publication. References of the retrieved studies were scrutinized to find additional articles that might not have been included in the results of these search strategies. The University Ethics Committee code was not applicable in this article.

## Results and Discussion

### Endemic mycoses

#### Primary “endemic” mycoses

Fungal mycoses cause disease in susceptible individuals irrespective of their immune status and are endemic which means they are found in specific geographical locations. These infections include histoplasmosis, blastomycosis, coccidioidomycosis, and paracoccidioidomycosis [ 6 ]. Incidence of both overt and subclinical human histoplasmosis caused by *H. capsulatum* and *Histoplasma duboisii* have been reported in Africa. Clinical manifestations of this disease are broad and can vary from asymptomatic disease or chronic cavitary pulmonary disease to life-threatening acute progressive disseminated histoplasmosis in patients with advanced HIV infection. 

Intradermal histoplasmin skin test (HST) is required for the determination of prior subclinical infections. In Africa, the prevalence rate of positive HST is within the range of 0-35% [ 7 ]. Almost 40% of cases of histoplasmosis in Africa occur in PLHIV with very high case fatality rates [ 8 ]. Diagnosis of acute disseminated histoplasmosis requires urinary *Histoplasma* antigen tests, histological examination, and cultures of the appropriate specimens, such as bone marrow and tissues from organs [ 9 ]. 

There has been no report of paracoccidioidomycosis or its causative agents, *Paracoccidioides braziliensis/ lutzii*, in Africa [ 6 ]. Several cases of blastomycosis, two cases of talaromycoses, and five cases of coccidiodomycosis have been documented across Africa, in West Africa, and in Uganda, respectively [ 6 , 10 ]. These endemic mycoses were diagnosed by culture and histopathological examinations [ 6 , 10 ]. 

#### Fungal neglected tropical diseases

The FNTDs consist of a diverse group of implantation mycoses which usually present much later after the initial infection. These diseases include sporotrichosis, mycetoma (Madura foot), chromoblasto-mycosis (chromomycosis), lacaziosis (lobomycosis), and entomophthoromycosis (subcutaneous zygomycosis). The causative organisms primarily affect the cutaneous and subcutaneous tissues; however, in most cases, they also involve adjacent structures, such as the lymphatic, cartilage, fascia, joints, and bones [ 11 ].

Mycetoma may be caused by a fungus (eumycetoma), a filamentous bacterium (actinomy-cetoma), or both. It is a chronic, progressive, and destructive inflammatory disease usually of the subcutaneous tissues, mostly caused by a fungus called Madurella mycetomatis. It is not a modifiable disease; therefore, the global burden of the disease is poorly defined [ 12 , 13 ]. 

Mycetoma has been found in many African countries within the “mycetoma belt”, like Sudan, Senegal, Nigeria, Mauritania, Kenya, Cameroon, Somalia, Tunisia, Niger, and Ethiopia [ 14 , 15 ]. Diagnosis is usually made by both histological and cultural methods. It is estimated that over 10,000 cases of mycetoma annually occur in Africa with the majority of documented cases being taken care of at the Mycetoma Research Centre in Khartoum, Sudan [ 14 , 15 ].

Chromoblastomycosis (CBM) is a fungal disease caused by melanised fungi, most especially Fonsecaea pedrosoi, and Cladophialophora carrionii [ 16 ]. The CBM lesions are usually polymorphic and should be distinguished from those associated with other benign or malignant clinical syndromes [ 16 ]. The true burden of this disease in Africa may be underestimated due to the paucity of epidemiological studies in the continent. It is noteworthy that Madagascar has the most cases of CBM in Africa [ 16 ]. 

Diagnosis of CBM requires laboratory confirmation by direct mycological examination and/or histopa-thology. To confirm the diagnosis of this disease, muriform cells in clinical specimens must be visualized [ 11 ]. Serological and intradermal tests have not been standardized for CBM and are not used in the routine laboratory. However, an Enzyme-linked immunosorbent assay diagnostic technique could be used which usually shows the existence of asymptomatic infection [ 17 ].

Sporotrichosis, caused by the Sporothrix schenckii complex is a chronic granulomatous mycosis acquired by traumatic inoculation among gardeners and farmers following pricks or scratches by thorns or other small injuries [ 18 , 19 ]. It must be noted that occasionally, traumatic exposure could be absent. Many occupational and leisure activities, like fishing, hunting, horticulturing, gardening, and farming, could facilitate exposure to the organism [ 20 ]. It is also considered an important zoonosis, especially in Brazil, where Sporothrix braziliensis is transmitted through cat scratches [ 18 ]. This disease has been reported regularly in South Africa since the first case was described in the early 20^th^ century [ 18 , 19 ]. 

Sporotrichosis was reported in Gauteng province that included Pretoria, Botswana, Mpumalanga, and North West provinces, especially among gold mine workers [ 18 , 19 ]. Several cases were reported in Egypt, Congo, Tanzania, Zimbabwe, Sudan, and Nigeria. Due to the few medical mycology laboratories in the continent, the accurate distribution of the disease in Africa has not been clearly mapped [ 18 , 19 ]. There are still challenges in the establishment of a diagnostics molecular microbiology laboratory in developing countries, especially in Africa [ 21 ].

Entomophthoromycosis is another group of subcutaneous zygomycosis caused primarily by Basidiobolus ranarum and Conidiobolus coronatus [ 22 ]. Cases of conidiobolomycosis infections have been reported in equatorial Africa [ 23 ]. It is usually diagnosed by microscopy and isolation of *C. coronatus* in culture. The isolates can be demonstrated by the macroscopic and microscopic morphologic characteristics suggestive of *C. coronatus* and agents of entomophthoromycosis [ 22 ]. Any delay in diagnosis leads to increased mortality which could be worse in sub-Saharan Africa [ 22 ].

Tinea capitis is also endemic among children in Africa; however, it is not officially recognized as an FNTD [ 24 ]. Tinea capitis is the most common infection of the scalp, skin, and hair caused by dermatophytes. Its clinical manifestations vary from mild scaling with little alopecia to large inflammatory and pustular plaques with extensive alopecia [ 25 ]. Tinea capitis is still a common infection with prevalence rates exceeding 40% in some communities in Africa [ 26 ]. 

In a recent systematic review, we found the pooled prevalence of tinea capitis to be about 23% with an estimated 138 million children being affected by this condition in Africa [ 27 ]. The infection is diagnosed in the laboratory by microscopic examination of the scalp hair and skin samples and their cultivation on the Sabouraud dextrose agar plates containing chloramphenicol with and without cycloheximide [ 28 ]. One of the newest methods is trichoscopy which is a useful and rapid method for the diagnosis of tinea capitis. The new diagnostic methods that vary from dermoscopy to molecular laboratory tests have been developed despite the fact that some of these facilities have not been incorporated into the routine practice in many African health centers [ 29 , 30 ]. 

### Epidemic mycoses

The HIV pandemic predominantly drives epidemic cases of fungal diseases in Africa [ 5 , 31 ]. Cryptococcosis, Pneumocystis pneumonia (PCP), oropharyngeal, and oesophageal candidiasis, and to some extent, disseminated histoplasmosis and emergomycosis are the main fungal diseases that occur among HIV-infected individuals in Africa. 

#### Cryptococcosis

Cryptococcosis, caused mainly by the yeast of the species Cryptococcus neoformans and Cryptococcus gattii, is among the commonest causes of meningitis among HIV-infected individuals in Africa [ 32 ]. Cryptococcal meningitis accounts for up to 15% of AIDS-related deaths worldwide. In sub-Saharan Africa, 162,500 (73% of global cases) and 135,900 (75% of global cases) individuals catch cryptococcal meningitis with an annual high mortality rate [ 33 ]. In addition, 4.0-15.0% of patients with HIV have asymptomatic cryptococcaemia which may progress into overt meningitis [ 33 ].

Cryptococcal antigen (CrAg) test is the mainstay of diagnosis for both asymptomatic cryptococcaemia and overt cryptococcal meningitis. This test is cheap, highly sensitive, and specific with a turnaround time of ~15 min allowing rapid and accurate point of care diagnosis. Blood culture and cerebrospinal fluid (CSF) cultures may be obtained in patients with relapses or in research settings. The CrAg screening is routine in many countries in Africa [ 5 ]. 

#### Pneumocystis pneumonia

The PCP caused by the opportunistic fungi Pneumocystis jirovecii remains an important cause of hospitalization and deaths among hospitalized PLHIV. From 1995 to 2015, PCP was diagnosed in about 15.0–4.0% of PLHIV, mainly in-patients, 22.4% of whom were in sub-Saharan Africa. About 18.8% of these patients died, and PCP accounted for 6.5% of them [ 34 ]. Over the years, the widespread use of ART and early diagnosis of HIV has led to a marked reduction in the incidence of PCP among PLHIV. However, the infection is not well documented in most African countries due to a lack of diagnostic facilities [ 35 , 36 ]. 

Treatment of PCP with high doses of cotrimoxazole is found to be effective; nevertheless, it has considerable toxicity. Concurrent administration of corticosteroids has been found to reduce PCP mortality in AIDS patients and accelerate the incidence of other opportunistic infections. 

Any delay in the initiation of appropriate antimicrobial treatment for PCP could lead to poor clinical outcomes [ 37 ]. Meanwhile, microscopy has been the mainstay for the diagnosis of PCP since *P. jirovecii* does not grow in culture in the laboratory. Detection of beta-D-glucan in blood could be useful due to its sensitivity, while not specific [ 38 ]. Alternative methods of PCP diagnosis, which are faster and more accurate, include Real-time polymerase chain reaction and immunofluorescence microscopy.

Sensitivity of PCR is about 15-20% better than classical staining (Giemsa, Diff-Quick, Gomori methenamine-silver or Toluidine O blue) [ 39 ]. Few of the samples with excellent diagnostic yield include induced and expectorated sputum, bronchoalveolar lavage, and nasopharyngeal aspirates [ 40 , 41 ]. In young children, only nasopharyngeal aspirates are able to provide a realistic sample for the PCR diagnosis [ 42 ]. 

#### Oral candidiasis

Oral candidiasis is the commonest opportunistic fungal infection among immunocompromised individuals that is observed in 7.6-75.0% of patients with HIV infection [ 43 ]. It must be noted that it has a higher prevalence among those with advanced HIV disease. Oral candidiasis is the commonest manifestation of HIV infection in Nigeria and other parts of Africa [ 44 ]. Moreover, oral candidiasis and oesophageal candidiasis often co-exist in patients with advanced HIV disease. 

Oesophageal candidiasis is an AIDS-defining illness seen in about 12% of patients with HIV/AIDS in sub-Saharan Africa [ 45 ]. Symptoms of oropharyngeal candidiasis include a burning sensation in the mouth, oral pain, dry mouth, throat pain, and impaired taste. [ 44 ]. Epidemiology of oral candidiasis is still unclear and there is a need for the accurate identification of the species which could be limited to Africa. *Candida albicans* is known to be the most common cause; however, non-*albicans* *Candida* species are increasingly being recognized as the cause of more than 30% of oral candidiasis cases [ 43 ]. 

It has been shown that *C. dubliniensis* is primarily associated with oral carriage and oropharyngeal infections in HIV-infected patients [ 46 ]. The *C. albicans* complex needs the application of accurate and reliable tests demanding DNA analysis, such as DNA amplification by PCR [ 46 ]. 

#### Disseminated histoplasmosis in HIV/AIDS

Patients with advanced HIV disease in areas endemic for histoplasmosis usually present with acute disseminated histoplasmosis (ADH) [ 47 , 48 ]. The ADH is one of the major AIDS-defining infections and a major killer of HIV-infected patients in South and Central America where it accounts for ~5,000-10,000 out of the estimated 24,000 annual deaths from AIDS [ 49- 51]. 

In the USA, the incidence of AIDS-associated histoplasmosis has declined significantly in the past few years due to ART [ 52 , 53 ]. Disseminated histoplasmosis and miliary tuberculosis are clinically very similar; therefore, it is difficult to differentiate between the two [ 54 ]. Moreover, histoplasmosis–tuberculosis co-infections are relatively common [ 55 ].

Epidemiology of histoplasmosis is not understood completely in Africa largely due to the lack of diagnostic tests and also the lack of awareness and under-recognition of the disease among clinicians [ 10 , 56 ]. However, an increasing number of cases of histoplasmosis are being reported among African patients with advanced HIV disease [ 57 ]. From 1952 to 2017, 470 cases of histoplasmosis were reported mainly in the West African (n=179, 38.1%) region and in those infected with HIV (n=178, 38%) [ 8 ]. 

In the immunocompromised person, *Histoplasma* polysaccharide antigen detection tests allow rapid diagnosis of disseminated histoplasmosis in urine, serum, bronchoalveolar lavage, and CSF samples before positive cultures can be identified [ 58 ]. The antigen concentration is greatest in urine and can be used to monitor the response to antifungal therapy and identify recurrent patients [ 59 ]. In AIDS patients with disseminated disease, *Histoplasma* antigen has been detected in 95-100% and 80% of urine and serum samples, respectively [ 53- 61]. 

### Emerging and re-emerging mycoses

Emerging fungal diseases are increasing due to the wider use of immunosuppressive medications, HIV pandemic, and the rapid rise of the number of immunocompromised patients with increased susceptibility to uncommon fungal pathogens [ 62 , 63 ].

#### Candidaemia and Candida auris

Invasive candidiasis is a life-threatening clinical condition that endangers critically ill patients and has a high fatality rate, ranging from 29% to 76% [ 64 ]. Recently, the incidence rate of invasive candidiasis caused by the non-*albicans* *Candida* species has undergone an increase [ 65 ]. Candidaemia and the rising incidence of resistance to antifungal medications pose an increasing threat to both the immunoco-mpromised and immunocompetent individuals, including cancer patients. The *C. albicans* is the species responsible for > 50% of invasive candidiasis globally. Diagnostic challenges could also arise from the effect of *C. albicans* hyphae and biofilm development. 

*Candida* bloodstream infections can cause significant mortality especially among the patients in the intensive care unit. The *C. albicans* remains the most frequently isolated *Candida* species in the clinical setting; however, there is an increase in the incidence rate of non-*albicans* *Candida* species infections in some countries, including sub-Saharan Africa [ 66 ].

The *C. auris* is an emerging species and a multidrug-resistant fungal pathogen that is associated with severe clinical disease. The *C. auris* infections have also been reported in Africa, especially Kenya and South Africa as early as 2009 [ 67 , 68 ]. Diagnosis of *C. auris* infections is usually confirmed by the conventional blood culture and antifungal susceptibility testing. However, despite its rapid global spread and the threat imposed by this species of *Candida*, it is still difficult to predict the actual burden of the infection [ 69 ]. In Africa, most pathology laboratories cannot fully identify the species level of all *Candida* isolates and the isolates were mostly reported as *Candida* species [ 70 ].

#### Blastomyces emzantsi

*Blastomyces dermatitidis*, *B. percursus*, and *B. emzantsi* are the causative agents of blastomycosis [ 71 ]. Extrapulmonary (skin or bone) disease, probably resulting from haematogenous spread from a primary lung infection, is the commonest presentation among the patients who were infected with these organisms.

#### Emergomycosis

*Emergomyces* is a dimorphic fungus with human pathogenic potential and consists of five known species that have been reported globally. These species include *Es. pasteurianus*, *Es. africanus*, *Es. canadensis*, *Es. Orientalis*, and *Es. europaeus*. *Es. pasteurianus* and *Es. africanus* are commonly isolated from Africa (Lesotho, Uganda, and South Africa) [ 72 , 73 ]. The earliest known isolate among these *Emergomyces* species was discovered in 1992. It remains unclear if these fungi have truly emerged or whether they are just recognized due to the increase in the number of susceptible hosts, improved microbiology capacity, and/or the widespread adoption of molecular identification techniques in clinical and research laboratories [ 74 , 75 ]. 

Majority of patients with these infections were immunocompromised [ 75 , 76 ]. Moreover, most patients with *Es. Africanus* infection usually have cutaneous lesions and pulmonary diseases [ 75 , 76 ]. Emergomycosis can be diagnosed through a biopsy of affected tissue for histopathology and fungal culture. When the fungal cultures are negative (or omitted), the PCR of the affected fresh tissue using internal transcribed spacer amplification and sequencing may establish the correct diagnosis [ 74 , 75 ]. 

#### Chronic pulmonary aspergillosis

Chronic pulmonary aspergillosis (CPA) is a slowly progressive and debilitating parenchymal lung disease occurring in patients with underlying lung diseases and subtle immunodeficiency [ 76 ]. Previously treated TB is the most common risk factor for CPA reported in 15-93% of cases [ 77 ]. Diagnosis of CPA requires a combination of clinical characteristics which include one or more cavities (with or without a fungal ball present) or nodules on thoracic imaging. Other diagnostics include direct evidence of *Aspergillus* infection (in microscopy or culture from biopsy) or immunological response to *Aspergillus* spp. and exclusion of alternative diagnoses. It should be noted that they should be present for at least three months [ 76 ]. 

Elevation of the *Aspergillus* antibody observed in over 90% of patients is the cornerstone for CPA diagnosis [ 76 ]. Culture of respiratory samples is insensitive and laborious. In addition, chest-imaging findings are not pathognomonic and may resemble TB or lung cancers. Based on the results of two epidemiological studies performed in Africa, CPA affects 5-10% of patients in their last month of TB treatment to two years after successful TB treatment [ 78 , 79 ]. Serological diagnosis of CPA is currently not possible in most parts of Africa due to the non-availability of the required kits.

#### Fungal asthma

Number of patients with Asthma is growing in Africa, and based on the findings of a few studies, fungal sensitization or allergic bronchopulmonary aspergillosis (ABPA) has been observed among this population group. Severe asthma with fungal sensitization is diagnosed among patients with bronchial asthma, positive type-1 skin prick test to *Aspergillus* allergens and/or raised *Aspergillus fumigatus*-specific IgE, negative (usually) *A. fumigatus* specific IgG, total IgE < 1000 IU/mL (usually less than 500 IU/mL), normal or central bronchiectasis in less than three lobes, no centrilobular nodules/mucoid impaction/hyperdense mucus, and eosinophil count generally < 500 cells/microliter [ 80 ].

Meanwhile, in the diagnosis of ABPA, the following criteria have to be met: 1) a predisposing underlying condition of either asthma or cystic fibrosis; 2) an elevated total IgE level greater than 1000 IU/mL and either a positive *Aspergillus* skin prick test or detectable *A. fumigatus*–specific IgE; and 3) at least two of the following three criteria of serum precipitating or *Aspergillus*–specific IgG antibodies, characteristic radiographic findings, and elevated total eosinophil count [ 81 ]. 

In a systemic review of studies published in Africa, the prevalence of fungal sensitization was relatively high (3–52%) in the asthmatic population with an average of 28% and a pooled estimate of 23.3%, mostly due to *Aspergillus* species. About 1.6–21.2% of patients with fungal sensitization had ABPA [ 82 ], the majority of whom had been diagnosed using a skin prick test. Serological detection of *Aspergillus*-specific IgG and IgE is scarce in Africa. 

#### Other fungal diseases

*Candida* vulvovaginitis or vulvovaginal candidiasis (VVC) is a typical infection in females which occurs primarily in women of reproductive age and could affect as many as one out of every two women [ 83 ]. Most women in their childbearing age develop VVC at least once in their lifetime [ 84 ]. The VVC is recognized with signs and symptoms of inflammation in the presence of a positive culture for *Candida* species and the absence of other infective agents [ 85 ]. Recurrent VVC (RVVC) is a clinical condition characterized by occurrences of VVC more than four times during a year in the presence of a recognizable risk factor [ 86 ]. 

According to the results of previous studies, RVVC was estimated to occur in 37,390 (3003/100,000 females) adult women in Namibia [ 87 ]. However, true population-based studies have not been conducted across Africa [ 88 ]. The diagnosis of VVC and RVVC is based on the clinical observation compatible with signs and symptoms that may or may not be pathogenically or clinically distinct and in most cases could represent the continuum of vaginal response to *Candida* species [ 89 ]. Most women with RVVC develop flare-ups without any identifiable risk factors which implies that genetic factors are likely to play an essential role in their susceptibility to RVVC. Diagnoses of genetic factors as well as antifungal resistance are still challenging in Africa. 

## Conclusion

Fungal diseases are common in Africa; however, the true burden of serious fungal diseases remains unknown. Based on the results, key drivers of fungal diseases in Africa were HIV/AIDs, tuberculosis, poverty, and the increasing number of patients with non-communicable diseases. 

Regarding the emerging fungal diseases, more advanced technologies, including whole-genome sequencing or simpler methods, such as Sanger sequencing, matrix-assisted laser desorption/ionization time-of-flight mass spectrometry, and histopathological and/or culture-based methods are required for species-level identification. These advanced diagnostics are not routinely performed in most centers across the continent. Lack of these diagnostics, and to some extent, the shortage of human resources in mycology makes accurate diagnosis of fungal diseases impossible. 

## Acknowledgments

None.

## Authors’ contribution

F.B. and S.A.F. contributed equally to this research project.

## Conflict of Interest: 

Not applicable.

## Financial disclosure

Not applicable.
